# Implant Health in Treated Periodontitis Patients: A Systematic Review and Meta-Analysis

**DOI:** 10.3390/dj12080240

**Published:** 2024-07-29

**Authors:** Léa Marty, Alain Hoornaert, Bénédicte Enkel, Alan Penhoat, Jacques Colat-Parros, Assem Soueidan, Fabienne Jordana

**Affiliations:** 1 Dental Faculty, Nantes University, 1 Place Alexis Ricordeau, BP 84215, 44042 Nantes, France; lea.marty17@hotmail.fr (L.M.); alain.hoornaert@univ-nantes.fr (A.H.); benedicte.enkel@univ-nantes.fr (B.E.); assem.soueidan@univ-nantes.fr (A.S.); 2 Dental Faculty, University of Bordeaux, 146 Rue Léo Saignat, 33076 Bordeaux, France; j.colat-parros@wanadoo.fr

**Keywords:** dental implants, periodontal diseases, systematic review

## Abstract

Background: The aim of this meta-analysis was to evaluate the role of a history of periodontitis on implant failure. The two main judgment criteria studied are peri-implantitis and the survival rate. The two secondary judgment criteria studied are the mean pocket depth and the mean peri-implant bone loss. Methods: An electronic search was performed via five databases (MEDLINE, Embase, ScienceDirect, LILACS and the Cochrane Library) and was supplemented by manual searching. The search was undertaken in June 2024. Results: Of 10 775 potentially eligible articles, 8 were included in the qualitative analysis and 10 in the quantitative synthesis. Conclusions: This meta-analysis suggests that a history of periodontitis has a significant impact on the rate of peri-implantitis, survival rate, mean bone loss and pocket depth.

## 1. Introduction

The prevalence of periodontal disease was reported to range from 20% to 50% around the world [1]. Upward of 1 in 10 adults worldwide may be affected by severe periodontitis [2]. Teeth affected by periodontitis can be preserved in the long term thanks to appropriate treatment and regular periodontal maintenance while respecting rigorous oral hygiene. The documented dental survival rate with these conditions is between 87% and 95% [3] after 10 years of maintenance and between 92% and 93% [4] after 50 years. However, during periodontitis, the advanced destruction of the attachment system of the dental organ, including bone resorption, can lead to the therapeutic decision to extract non preservable teeth, representing between 30% and 35% [5] of all dental extractions.

The implant has become a treatment of choice to replace missing teeth, whatever the etiology, in totally or partially edentulous patients [6,7]. Implants have a satisfactory survival rate after 10 years, between 82% and 94% [4], but are not exempt from complications. One of the most important is inflammation of the peri-implant tissues [8]. Peri-implant health is defined by the absence of clinical signs of inflammation, bleeding or suppuration during delicate probing, the absence of an increase in probing depth compared to previous examinations and the absence of bone loss [9]. Peri-implant diseases are inflammatory diseases of the soft and/or hard tissues juxtaposed to the implants. They are classified into two groups: peri-implant mucositis and peri-implantitis [10]. The use of dental implants has radically changed the way to partially and totally rehabilitates edentulous patients, thus allowing clinicians to perform complex oral rehabilitations [11,12]. Some studies have reported on the potential association between history of periodontitis and peri-implantitis [13,14,15,16]. It would be interesting to carry out a meta-analysis to bring together all the relevant published data and analyze them statistically.

The aim of this review was to evaluate the role of a history of periodontitis on implant health in humans through a meta-analysis. This meta-analysis enables us to study the influence of initial periodontal diagnosis on implant health.

## 2. Materials and Methods

The protocol of this study was designed according to the guidelines [17,18] and was registered in the National Institute for Health Research PROSPERO database (CRD42022371232). The specific PECO (population, exposure to risk factor, comparisons and outcomes) framework used to devise the focused question is described below. The study protocol was designed to compare the results of implant health in patients with a history of periodontal disease with those in healthy patients without a history of periodontal disease. The following criteria were studied: the rate of peri-implantitis, implant survival and the level of bone loss and pocket depth.

### 2.1. Inclusion Criteria

1. Randomized clinical trials, case–control and cross-sectional studies and prospective and retrospective cohort studies in humans.
2. Only studies published in English in an international peer-reviewed journal.
3. The study population must be between 18 and 80 years old.
4. All patients with periodontitis should have received prior nonsurgical or surgical periodontal treatment as needed. Periodontitis must have been inactive during the study. The subjects must also have been educated in correct and rigorous oral hygiene and continuing supportive periodontal maintenance. Clinically, implant follow-up after loading should have been performed for more than 2 years.

### 2.2. Exclusion Criteria

1. Case report studies, systematic reviews and veterinary clinical trials
2. Any study including patients with serious pathologies that could affect implant therapy
3. Publications considering zygomatic, transmandibular implants and orthodontic temporary anchorage implants
4. Studies in which the surgical intervention mentions immediate implantation or immediate prosthetic loading
5. If the number of subjects included, N, was strictly less than 15, the power of the study was considered too weak.
6. Concerning attrition, if the rate of loss to follow-up was greater than or equal to 20% according to the standards of the Cochrane collaboration, the bias was considered too great to select the study.

Based on a comprehensive strategy, MEDLINE, LILACS, Embase, ScienceDirect and Cochrane Library databases were searched to identify eligible articles published in the English language up to June 2024. Additional manual searching of reference lists in the articles selected and in a number of review articles was performed.

The following keywords were used for this purpose:

Treated periodontitis OR aggressive periodontitis OR chronic periodontitis

AND dental implant OR oral implant

Quality assurance was developed by independent screening according to Khan et al. [19]. When disagreement arose in the selection and eligibility, it was resolved by discussion between the 2 reviewers (L.M., F.J.).

The assessment of methodological quality and risk of bias of each included publication was performed [20].

This review included 18 studies, 10 in the statistical analysis (Table 1 [13,14,15,16,21,22,23,24,25,26,27,28,29,30,31,32,33,34], Supplementary Table S1 [35,36,37,38,39,40,41,42,43,44,45,46], Supplementary Figure S1 [14,15,21,22], Figure S2 [13,23,24,25,26] and Figure S3 [27,32,34]) and 8 in the quantitative analysis (Supplementary Figure S4 [16,28,29,30,31] and Figure S5 [33]), with an overall low risk of bias.

In the five cross-sectional studies, two publications presented a low risk of bias with 100% of the positive items: Sayardoust et al. [28] and Meyle et al. [29]. One publication, Pandolfi et al. [31], reported a low risk of bias with 87.5% of positive items. Two publications presented a low risk of bias with 75% of positive items: Di Guarnieri et al. [30] and Aguirre et al. [16]. The RCT by Raes et al. [33] reported a low risk of bias, with 70% of positive items. Case-control studies of Cho-Yan Lee et al. [27], Vagia et al. [32] and Xu et al. [34] presented a low risk of bias with 100% of positive items. Among the cohort studies, four publications presented a low risk of bias with 100% of positive items [13,21,24,26]. Four publications [14,15,23,25] presented one item with unclear information, and one publication [22] presented one negative item.

**Figure 1:**
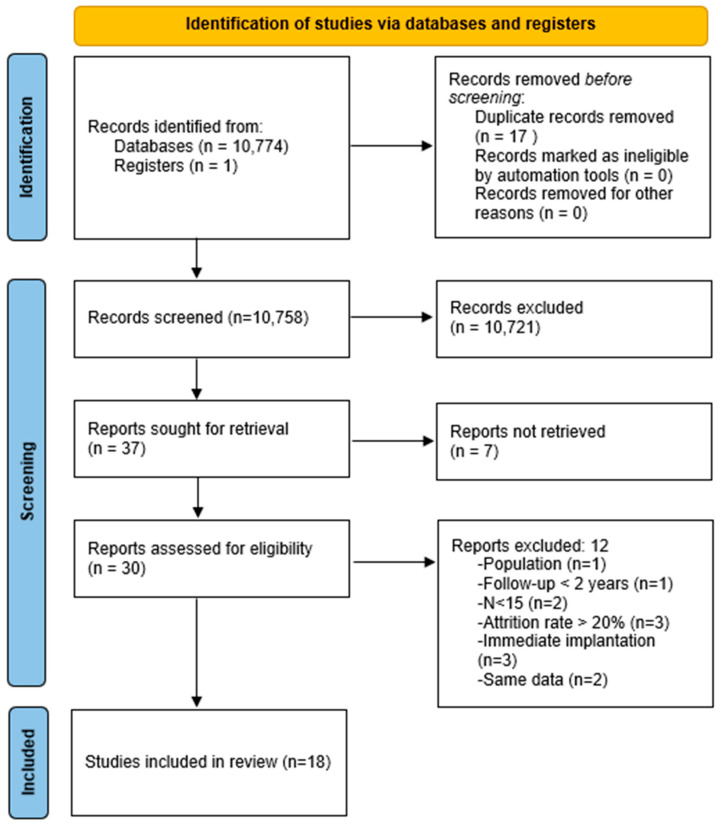
Preferred reporting Items for Systematic Reviews and Meta-Analysis (PRISMA 2020) flow diagram demonstrating the results of the systematic literature search.

The search yielded 10 775 results. Eighteen studies fulfilled the inclusion criteria (Figure 1).

Data were collated into tables (Table 1, Table 2 and Table 3) regarding the population, the intervention and the outcomes.

Heterogeneity between studies was assessed using Cochran Q-statistics and the I2-measure. I2-values of 25–49% were considered to indicate low, 50–74% as moderate and ≥75% as high levels of heterogeneity (Supplementary Figures S6–S9).

Statistical analysis was carried out using Comprehensive Meta-Analysis 3.0 software. Subgroup analyses or pairwise meta-analyses were also performed if applicable to explore the data further. In this context, *p* < 0.05 indicated a significant difference.

## 3. Results

### 3.1. Study Selection

The initial search of the literature up to June 2024 yielded 1775 potentially suitable articles. The design of the included studies of PRISMA selection is reported in Supplementary Table S2 [13,14,15,16,21,22,23,24,25,26,27,28,29,30,31,32,33,34] in the Supplementary Materials. A meta-analysis was performed on 10 studies [13,14,15,21,22,23,24,25,26,27] that compared groups of healthy patients and groups of patients with a history of periodontitis. Ten studies were included in the quantitative synthesis. A meta-analysis could not be performed on eight studies [16,28,29,30,31,32,33,34], which did not compare patients with a history of periodontitis with healthy patients. These eight studies were included in the qualitative synthesis. The κ value for interviewer agreement for study inclusion was 0.93 for titles and abstracts and 1.00 for full-text articles, indicating strong agreement.

### 3.2. Study Characteristics

In the quantitative synthesis, a total of 1571 patients and 4030 implants were included in this review.

In the qualitative synthesis, a total of 2504 patients and 6552 implants were included in this review.

### 3.3. Quantitative Synthesis

Concerning population and intervention characteristics, in all studies, the diagnosis of periodontitis was established following the Armitage definition of 1999 [47]. Each patient with periodontal disease underwent nonsurgical periodontal treatment prior to implant surgery. In some cases, surgical periodontal treatment was necessary.

#### 3.3.1. Peri-Implantitis Rate

The most significant results, described below, are summarized in Table 4.

-Periodontitis group vs. Healthy group:

For the statistical analysis of the groups of the five articles [13,14,24,26,27] studying the rate of peri-implantitis in the quantitative synthesis, Supplementary Figure S10 [13,14,24,26,27] compares the rate of peri-implantitis in patients with a history of periodontitis, with all diagnostics combined, compared to healthy patients. The overall result indicates a 4.8-fold higher risk, over a follow-up period between 8 and 16 years, of developing peri-implantitis among patients with a history of periodontal diseases than in healthy patients, with a statistically significant difference, OR = 4.80 (95% CI = 3.29–7.00), *p* < 0.05.

-Aggressive periodontitis group vs. Healthy group:

In Supplementary Figure S10 [13,14,24,26,27], the generalized aggressive periodontitis group, studied in the article by Swierkot et al. [24], had a six times higher risk of developing peri-implantitis than the group of healthy patients, OR = 6.00 (95% CI = 1.19–30.17), with a statistically significant difference, *p* < 0.05. This study highlights an association, not due to chance, over a period of 16 years between the occurrence of peri-implantitis and exposure to a history of generalized aggressive periodontitis.

-Aggressive periodontitis group vs. Healthy group:

The results of the four studies [13,14,26,27] testify to an association, not due to chance, between the occurrence of peri-implantitis and exposure to a history of chronic periodontitis without distinction of severity compared to the group of healthy patients. Statistical analysis of these different studies leads to the conclusion that there is an approximately 4.6 times higher risk of developing peri-implantitis with a history of chronic periodontitis over a follow-up period of between 8 years and 14 years, with a significant difference: OR = 4.55 (95% CI = 2.97–6.98), *p* < 0.05 (Supplementary Figure S11 [13,14,26,27]).

-Chronic periodontitis group vs. Aggressive periodontitis group:

An odds ratio of 1.59 (95% CI = 0.94–2.69) with a *p* > 0.05 (Supplementary Figure S12 [13,14,26,27]) means that there is no significant difference between the rate of peri-implantitis in patients with a history of CP and in patients with a history of AP.

The least significant results are described below because only two studies could be used.

By combining the data from the two studies by Roccuzzo et al. from 2010 and 2014 [13,26], it is possible to conclude that there is an association, not due to chance, over a follow-up period of 10 years between the occurrence of peri-implantitis and exposure to a history of severe chronic periodontitis compared to the group of healthy patients, OR = 8.17 (95% CI = 3.50–19.10), *p* < 0.05, (Supplementary Figure S13 [13,26]).

By combining the data from the two studies [13,26], it is possible to conclude that there is an association, not due to chance, over a follow-up period of 10 years between the occurrence of peri-implantitis and exposure to previous moderate chronic periodontitis compared to the group of healthy patients, OR = 4.05 (95% CI = 1.74–9.42), *p* < 0.05 (Supplementary Figure S14 [13,26]).

It is possible to conclude that there is an association, not due to chance, between the occurrence of peri-implantitis and exposure to a previous SCP over a follow-up period of 10 years. The risk was approximately twice as high compared to the group of patients with a history of MCP, OR = 0.48 (95% CI = 0.26–0.92), *p* < 0.05 (Supplementary Figure S15 [13,26]).

#### 3.3.2. Survival Rate

The most significant results, described below, are summarized in Table 4.

-Periodontitis group vs. Healthy group:

The overall result shows that the implant survival rate is 1.7 times higher over a follow-up period from 2 to 16 years in healthy patients than in patients with a history of periodontitis, regardless of the periodontal diagnosis. The difference was statistically significant, OR = 0.60 (95% CI = 0.44–0.83), *p* < 0.05 (Supplementary Figure S16 [13,15,21,22,23,24,25,26]).

-Chronic periodontitis group vs. Healthy group:

Statistical analysis of the results of the six studies [13,15,21,23,25,26], involving groups of patients with a history of chronic periodontitis without distinction of severity over a follow-up period from 2 to 12 years, resulted in a significant difference in the survival rate of 1.7 times higher in healthy patients, OR = 0.60 (95% CI = 0.41–0.89), *p* < 0.05 (Supplementary Figure S17 [13,15,21,23,25,26].).

-Chronic periodontitis group vs. Aggressive periodontitis group:

In conclusion, an odds ratio of 0.55 (95% CI = 0.38–0.80) with a *p* < 0.05 indicates a significant difference between the survival rate in patients with a history of CP and in patients with a history of AP (Supplementary Figure S18 [13,15,21,23,25,26]).

The least significant results are described below because only two studies could be used.

The statistical analysis of the two studies concerned [22,24], with an odds ratio of 0.44 (95% CI = 0.06–3.46) with *p* > 0.05, means that the survival rate is 2.3 times higher in healthy patients than in patients with a history of AP, without excluding the probability of chance in these results. There was no significant difference. The implant failure rate increases slightly between 4 and 8 years of follow-up in patients with a history of AP (Supplementary Figure S19 [22,24]).

It is possible to conclude that the survival rate is substantially the same in the groups of patients with a history of MCP and the groups of healthy patients over a period of follow-up from 10 to 12 years, OR = 0.78 (95% CI = 0.43–1.42), *p* > 0.05 (Supplementary Figure S20 [13,23,26]). Over a period of more than 10 years, the survival rate was 1.9 times higher in healthy patients than in patients with a history of severe chronic periodontitis, with a significant difference, OR = 0.54 (95% CI = 0.34–0.86), *p* < 0.05 (Supplementary Figure S21 [13,23,26]).

The overall statistical analysis of the three studies [13,23,26] described an OR of 1.46 (95% CI = 0.90–2.37) with a *p* > 0.05. This means that there was no significant difference in the survival rate depending on the severity of chronic periodontitis over a follow-up period from 10 to 12 years (Supplementary Figure S22 [13,23,26]).

#### 3.3.3. Mean Peri-Implant Bone Loss

The most significant results, described below, are summarized in Table 4.

In the overall statistical analysis of these three studies [13,21,27], over a follow-up period from 10 to 14 years, the standardized mean difference in peri-implant alveolysis was 0.77 mm (±0.22 mm; *p* < 0.05), with a statistically significant difference (Supplementary Figure S23).

The least significant results are described below because only two studies could be used.

Over a period of 10 years, the average standardized difference between the group of patients with a history of PCM and the group of healthy patients concerning alveolysis was statistically significant, with a value of 0.38 mm (±0.17 mm; *p* < 0.05) (Supplementary Figure S23 [13,21,27]).

The standardized mean difference between the group of patients with a history of SCP and the group of healthy patients concerning alveolysis was statistically nonsignificant, with a value of 0.21 mm (±0.17 mm; *p* > 0.05) (Supplementary Figure S23 [13,21,27]).

The standardized difference in means between the groups of patients with a history of MCP and SCP concerning alveolysis was statistically non-significant, with a value of 0.14 mm (±0.15 mm; *p* > 0.05) (Supplementary Figure S24 [24]).

#### 3.3.4. Mean Pocket Depth

The most significant results, described below, are summarized in Table 4.

Regarding the results of the average pocket depth, in patients with a history of periodontitis, regardless of the diagnosis, over a follow-up period from 4 to 14 years, the Hedges’ “s” was 0.49 (±0.18, *p* < 0.05). There was an average effect of a history of periodontitis on the mean peri-implant PDP compared to healthy patients, with a significant difference (Supplementary Figure S25 [13,15,22,26,27]).

In the overall statistical analysis of these four studies [13,18,26,27], over a follow-up period of 5, 10 and 14 years, the standardized mean difference concerning the peri-implant PPD had a value of 0.56 mm (±0.20 mm; *p* < 0.05), with a statistically significant difference (Supplementary Figure S26 [13,15,26,27]).

The standardized difference in means between the groups of patients with a history of CP and AP concerning PPD was statistically non-significant, with a value of 0.44 mm (±0.32 mm; *p* > 0.05) (Supplementary Figure S27 [13,15,26,27]).

The least significant results are described below because only two studies could be used.

The standardized mean difference between the group of patients with a history of AP and the group of healthy patients concerning the depth of pockets was statistically insignificant, with a value of 0.11 mm (±0.20 mm; *p* > 0.05). The Hedges’ “g” of 0.11 (±0.20 mm; *p* > 0.05) resulted in a weak effect of the history of AP on the mean peri-implant PPD compared to healthy patients, with no statistically significant difference (Supplementary Figure S28 [22]).

Two articles [13,26] studied groups of patients with a history of moderate chronic periodontitis over a period of 10 years. The standardized mean difference between the group of patients with a history of SCP and the patients with peri-implant PPD was statistically significant, with a value of 0.34 mm (±0.18 mm; *p* < 0.05) (Supplementary Figure S29 [13,26]).

The same two articles [13,26] studied groups of patients with a history of severe chronic periodontitis over a period of 10 years. The standardized difference in means between the group of patients with a history of SCP and the group of healthy patients concerning the peri-implant PPD was statistically insignificant, with a value of 0.79 mm (±0.48 mm; *p* > 0.05) (Supplementary Figure S30 [13,26]).

The standardized difference in means between the groups of patients with a history of MCP and SCP concerning PPD was statistically nonsignificant, with a value of 0.32 mm (±0.17 mm; *p* > 0.05) (Supplementary Figure S31 [13,26]).

### 3.4. Qualitative Synthesis

Concerning population and intervention characteristics, in all studies, the diagnosis of periodontitis was established following the Armitage definition of 1999 [47]. Each patient with periodontal disease underwent nonsurgical periodontal treatment prior to implant surgery. In some cases, surgical periodontal treatment was necessary.

#### 3.4.1. Peri-Implantitis Rate

At 5 years, Raes et al. [33], Meyle et al. [29] and Aguirre et al. [16] described, respectively, a peri-implantitis rate of 7.14%, 8.9% and 8%. At 10 years, Pandolfi et al. [31] and Vagia et al. [32] described a similar peri-implantitis rate of 12.9% and 12.8%, respectively.

#### 3.4.2. Survival Rate

Sayardoust et al. [28], Meyle et al. [29], Raes et al. [33], Di Guarnieri et al. [30], Pandolfi et al. [31] and Xu et al. [34] described, respectively, a survival rate of 92.9%, 96.3%, 97.3%, 90%, 96% and 95.4%.

#### 3.4.3. Mean Peri-Implant Bone Loss

The mean bone loss seems higher in patients with treated aggressive periodontitis at 5 years with moderately roughened implants than in patients with a history of chronic periodontitis [28,29,33]. Mean alveolysis does not seem to be higher in patients with treated aggressive periodontitis at 5 years for low-roughness implants than in patients with treated chronic periodontitis. On the other hand, tobacco seems to have an impact. Concerning the alveolysis mean, it seems preferable in patients with a history of chronic periodontitis to place implants with moderate roughness in nonsmokers (*p* < 0.05) [28]. At 10 years, in patients with treated periodontitis, it seems that the alveolysis mean increases if there is tobacco consumption in the population [28,30].

#### 3.4.4. Mean Pocket Depth

At 5 years, there was no significant difference between the mean PPD of low-roughness implants and moderate roughness implants in patients with treated AP [29,33]. The mean PPD was slightly lower (from 0.2 mm to 1.3 mm) in patients with a history of PC compared to patients with a history of AP over a period of 5 years [29,33]. There was a significant difference in the evolution of the mean PPD over time, with the mean PPD at 10 years being significantly higher than the mean PPD at 5 years (*p* < 0.05) [29].

## 4. Discussion

The results of the qualitative synthesis seem to vary for the same type of periodontal diagnosis. For a history of CP, over the same loading period of 10 years and with the same implant surface roughness, the rate of peri-implantitis can vary from 12.9% [31] to 23.8% [29] according to studies. This variation may be explained by the fact that the studies did not use exactly the same parameters to diagnose peri-implantitis. Indeed, the severity of periodontitis was not always specified and discriminated in studies, although its information is an integral part of a precise periodontal diagnosis. Moreover, the two studies did not have the same implant population, with N = 1991 [31] and N = 54 [29]. If the rate of PI seems to increase over time, it is impossible to conclude that there is a real association. Indeed, the study by Aguirre et al. [16], following the calculation of the odds ratio, discerned the absence of an association between the duration of follow-up and the PI rate (*p* > 0.05). It also did not distinguish any significant difference, at the level of the rate of PI, between the groups of chronic and aggressive periodontitis. This result is consistent with the conclusion of our meta-analysis. There does not seem to be a significant difference in the rate of PI between patients with a history of AP and CP. The rate of peri-implantitis is higher with greater implant roughness [29,33]. On the other hand, if the roughness seems to influence the rate of peri-implantitis, the aggressiveness or the chronicity of the periodontitis does not seem to have a clear link of association with the rate of PI in these articles [25,28,29,33]. Similarly, if the PI rate seems to increase over time, it is impossible to conclude that there is a real association. The 2018 systematic review by Jordana et al. [48] on the rate of peri-implantitis and different implant roughness asserted the same conclusion with a significant difference. There is an association between peri-implantitis and duration of follow-up only for significant implant roughness [48]. The 2022 retrospective study by Lombardo et al. [49] asserted that peri-implantitis prevalence was 7.84% in periodontal patients with excessive bone loss at 1 mm after 5 years. This result is in line with the results of our analysis.

At 5 years, the survival rate was found to be higher for implants with moderate roughness compared to implants with low roughness [28]. Regarding patients with a history of CP, it seems more interesting to place implants with moderate roughness in smokers in order to benefit from the higher survival rate compared to implants with low roughness. On the other hand, in nonsmokers, the two types of implants have approximately the same survival rate. The study by Xu et al. [34] concluded that periodontal status did not significantly affect the early implant survival rate but increased the risk of late implant loss.

In patients with a history of AP, it seems more interesting to place moderately rough implants, with a higher survival rate compared to low roughness implants [33]. At 10 years, concerning patients with a CP history with implants with a moderately rough surface, the mean survival rate is 94.1% [29,30,31].

In patients with a history of periodontitis, putative periodontal pathogens appeared to predominate in the microbiome of disease implant tissues, which confirms previous observations of periodontitis-associated species in deepened pockets around implants [40]. However, in the literature, the systemic review by Montenegro et al. [50] in 2020 stated that the majority of studies published to date have not shown any significant microbiome difference between chronic and aggressive periodontitis. This similarity in the microbiome may be part of the plausible explanations. Cortelli et al. (2012) found that the bacterial frequency tended to be higher in peri-implantitis and periodontitis sites than in healthy peri-implant and periodontal sites [51]. However, in the systematic review by Rakic et al., 2016 [52], considering the reviewed studies as a whole, the microbiologic profile in peri-implantitis is complex, variable and consists of Gram-negative anaerobic periopathogens. The presence of titanium seems to create a distinct microenvironment, and as a consequence, the microbiologic profile in peri-implantitis remains different from that of periodontitis. In perspective, further research could be conducted on this possible link between the microbiomes of different periodontitis and peri-implantitis cases.

In the results of our meta-analysis, the severity of periodontitis did not seem to influence the survival rate, and there was no significant difference between patients treated with MCP and SCP. On the other hand, the survival rate of implants in patients with treated SCP was significantly lower than that of implants in healthy patients. Further studies could be conducted to exploit this difference in results. Implants in patients with treated chronic periodontitis had a significantly lower survival rate than implants in patients with a history of aggressive periodontitis. In our qualitative synthesis, two studies described research on the survival rate of two types of implants, low and moderate roughness, in patients with a history of aggressive and chronic periodontitis. It would be interesting to conduct further research on this subject. There were some confounding factors in this meta-analysis like the heterogeneity of studies included. Some results of quantitative synthesis are less significant because only two or three studies could be used. Some diagnostic groups of periodontitis are poorly represented and require more data. Perhaps future publications will complement our work. It might be interesting to study the smoker and non-smoker population separately.

Regarding our meta-analysis, we were unable to compare patients with a history of chronic and aggressive periodontitis because none of the included articles studied the mean alveolysis in patients with treated aggressive periodontitis. One perspective would be to deepen research on the comparison of these two groups. Our results, concerning the influence of the severity of periodontitis on the mean bone loss, show a significant difference between the groups of patients. On the other hand, we did not find any significant difference between the groups of patients with a history of MCP and SCP between healthy patients and patients with treated SCP. The severity does not seem to influence the mean bone loss; however, further research is needed to confirm these results.

## 5. Conclusions

This meta-analysis suggests that a history of periodontitis significantly impacts the rate of peri-implantitis, survival rate, mean bone loss and pocket depth. The severity of periodontitis seems to influence the rate of peri-implantitis. However, the chronicity or aggressiveness of the treated periodontitis did not seem to influence the rate of peri-implantitis (*p* > 0.05). The severity of periodontitis does not seem to influence the survival rate. However, the chronicity or aggressiveness of the treated periodontitis seems to influence the survival rate. According to the results of our meta-analysis, over a follow-up period from 10 to 14 years, it seems that the history of periodontitis in general has a significant impact, with a large effect on the mean peri-implant bone loss. Over a follow-up period from 4 to 14 years, it seems that the history of periodontitis in general has a significant impact, with a medium effect on the mean of depth pockets.

According to research published in the literature in the last 10 years, implant placement is possible in patients with a history of periodontitis if the periodontal disease is well controlled and stabilized. The risk of developing implant health problems is still higher compared to healthy patients.

## Figures and Tables

**Table 1:** Design of the included studies of the PRISMA selection [13,14,15,16,21,22,23,24,25,26,27,28,29,30,31,32,33,34].

Name of the Study	Follow-Up Time	Type of Study	Inclusion
CASADO et al., 2013 [14]	8 years	Retrospective cohort studies	Quantitative synthesis
RASPERINI et al., 2014 [21]	10 years		
THÖNE-MÜHLING et al., 2016 [22]	4 years		
GRAETZ et al., 2017 [15]	10 years		
ROCCUZZO et al., 2010–2012 [13]	10 years	Prospective cohort studies	
LEVIN et al., 2011 [23]	12 years		
SWIERKOT et al., 2012 [24]	16 years		
JIANG et al., 2013 [25]	2 years		
ROCCUZZO et al., 2014 [26]	10 years		
CHO-YAN LEE et al., 2011 [27]	14 years	Retrospective case-control studies	
SAYARDOUST et al., 2013 [28]	5 years	Transversal studies	Qualitative synthesis
AGUIRRE et al., 2015 [16]	17 years		
MEYLE et al., 2014 [29]	10 years		
DI GUARNIERI et al., 2020 [30]	10 years		
PANDOLFI et al., 2020 [31]	10 years		
VAGIA et al., 2021 [32]	3 years	Retrospective study	
RAES et al., 2018 [33]	5 years	Clinical randomized trials	
XU et al., 2023 [34]	5 years	Retrospective study	

**Table 2:** Population and intervention characteristics, main and secondary outcomes of studies included in the quantitative synthesis [13,14,15,21,22,23,24,25,26,27].

	Population Characteristics				Intervention Characteristics	Main Outcomes		Secondary Outcomes	
Inclusion	Name of the Study	Periodontal Diagnosis Groups	Patients Number (N)	Implants Number	Type of Implant: Surface	Peri-Implantitis Rate (%)	Survival Rate (%)	Mean of Peri-Implant Bone Loss (mm)	Mean of Pocket Depth (mm)
Quantitative synthesis	Roccuzzo et al., 2010–2012 [13]	MCP	38	95	Straumann: TPS	27	92.8	1.14 ± 1.11	3.5 ± 0.9
		SCP	42	90		47.2	90	0.98 ± 1.22	3.9 ± 0.7
		H	32	61		10.7	96.6	0.75 ± 0.88	3.1 ± 0.5
	Cho-Yan Lee et al., 2011 [27]	CP	30	56	Straumann: SLA or TPS	36.7%		0.45 ± 0.94	2.83 ± 0.59
		H	30	61		16.7%		0.26 ± 0.72	2.81 ± 0.49
	Levin et al., 2011 [23]	MCP	149	447	Unclear		96.6		
		SCP	285	747			94.8		
		H	283	747			96.9		
	Swierkot et al., 2012 [24]	AP	35	149	Branemark MKII Nobel Biocare Osseotite Biomet 3i	43	96		
		H	18	30		11	100		
	Casado et al., 2013 [14]	CP	215	754	EH/IH/CM	59			
		H				27			
	Jiang et al., 2013 [25]	CP	30	149	Unclear		95.97		
		H	30	127			97.6		
	Rasperini et al., 2014 [21]	CP/Sm	60	120	Branemark: Machined		85	3.47 ± 1.09	
					Straumann: TPS			3.77 ± 1.43	
		CP/NSm			MS and TPS		90	2.32 ± 0.41	
		H/Sm	60		MS		95	2.65 ± 0.41	
					TPS			2.51 ± 0.31	
		H/NSm			MS		95	1.43 ± 0.38	
					TPS			1.95 ± 0.42	
	Roccuzzo et al., 2014 [26]	MCP	46	96	Straumann: SLA	52.2	96.9		4.6 ± 3.1
		SCP	45	102		66.7	97.1		4.8 ± 1.4
		H	32	54		18.8	100		4.4 ± 1.1
	Thöne-Mühling et al., 2016 [22]	AP	35	149	Branemark MKII Nobel Biocare Osseotite Biomet 3i		97.3		3.5 ± 0.7
		H	18	30			100		3.42 ± 0.81
	Graetz et al., 2017 [15]	CP	29	69	Unclear		92.5		4.2 ± 1.6
		H	29	76			91.4		2.9 ± 0.8
Total			1571	4030					

**Legends:** AP: aggressive periodontitis; CM: cone morse; CP: chronic periodontitis; EH: external hexagon; H: health; MS: machined surface; IH: internal hexagon; MCP: moderate chronic periodontitis; NR: not reported; NSm: nonsmokers; Sm: smokers; SCP: severe Chronic periodontitis; SLA: sand blast, large grit, acid-etch (implant surface); TPS: titanium plasma sprayed (implant surface).

**Table 3:** Population, intervention characteristics and main outcomes of studies included in the qualitative synthesis [16,28,29,30,31,32,33,34].

	Population Characteristics				Intervention Characteristics	Main Outcomes		Secondary Outcomes	
Inclusion	Name of the Study	Periodontal Diagnosis Groups	Patients Number	Implants Number	Type of Implant	Peri-Implantitis Rate (%)	Survival Rate (%)	Mean of Peri-Implant Bone Loss (mm)	Mean of Pocket Depth (mm)
	Sayardoust et al., 2013 [28]	SCP/Sm	80	252	Branemark: Machined TiUite: Oxidized	-	92.9	1.39 ± 1.57	-
		SCP/NSm						1.01 ± 1.09	
	Aguirre et al., 2015 [16]	CP	170	786	Astra Tech Nobel replace Steri-Oss	5 years: 8 10 years: 9 17 years: 15	-	-	-
		AP	69						
	Meyle et al., 2014 [29]	CP	20	54	Frialit 2 Dentsply	5 years: 8.9 10 years: 23.8	96.3	5 years: 0.23 ± 0.34 10 years: 0.63 ± 0.26	5 years: 2.9 ± 0.8 10 years: 3.3 ± 1.0
	Raes et al., 2018 [33]	AP	18	84	Branemark: Machined	5 years: 7.14	97.6	5 years: 1.00 ± 0.90	5 years: 3.1 ± 1.0
					TiUnite: Oxidized	5 years: 28.57	100	5 years: 1.65 ± 1.65	5 years: 4.2 ± 2.6
	Di Guarnieri et al., 2020 [30]	CP	58	127	-	-	90	3.1 ± 1.2	1–4 mm: 68.9 5–6 mm: 20.6 >6 mm: 10.5
	Pandolfi et al., 2020 [31]	CP	475	1991	Straumann	10 years: 12.9	96.1	-	-
	Vagia et al., 2021 [32]	CP	86	260	Srtaumann	10 years: 12,8	-	-	-
	Xu et al., 2023 [34]	MCP	1528	2998	Nobel Biocare	-	95,4%	-	-
Total			2504	6552					

**Legends:** AP: aggressive Ppriodontitis; CP: chronic periodontitis; H: health; M: machined; MCP: moderate chronic periodontitis; NR: not reported; NSm: nonsmokers; Sm: smokers; SCP: severe chronic periodontitis; SLA: sand blast; arge grit, acid-etch (implant surface); TPS: titanium plasma sprayed (implant surface).

**Table 4:** Most significant results on the meta-analysis.

	Diagnosis of Periodontitis	Odds Ratio	*p*-Value
Peri-implantitis rate	P group vs. H group	4.80	*p* < 0.05
	AP group vs. H group	6.00	*p* < 0.05
	CP group vs. H group	4.55	*p* < 0.05
	CP group vs. AP group	1.59	*p* < 0.05
Survival rate	P group vs. H group	0.60	*p* < 0.05
	CP group vs. H group	0.60	*p* < 0.05
	CP group vs. AP group	0.55	*p* < 0.05
		**Std diff in means**	
Mean PI bone loss	P group vs. H group	0.77 mm	*p* < 0.05
Mean pocket depth	P group vs. H group	0.56 mm	*p* < 0.05
	CP group vs. AP group	0.44 mm	*p* > 0.05

**Legends:** AP: aggressive periodontitis; CP: chronic periodontitis; H: health; MCP: moderate chronic periodontitis; P: periodontitis; PI: peri-implant; SCP: severe chronic periodontitis.

## Data Availability

The original contributions presented in the study are included in the article and Supplementary Material, further inquiries can be directed to the corresponding author.

## Supplementary Materials

The following supporting information can be downloaded at: https://www.mdpi.com/article/10.3390/dj12080240/s1, Figure S1: Results of heterogeneity tests, regarding the peri-implantitis rate of studies included in the quantitative synthesis [14,15,21,22]; Figure S2: Results of heterogeneity tests, regarding the survival rate of studies included in the quantitative synthesis [13,23,24,25,26]; Figure S3: Results of heterogeneity tests, regarding the alveolysis mean of studies included in the quantitative synthesis [27,32,34]; Figure S4: Results of heterogeneity tests, regarding the alveolysis mean of studies included in the quantitative synthesis [16,28,29,30,31]; Figure S5: Graph regarding bias assessment of analytic retrospective cohort studies using the Newcastle-Ottawa Scale (NOS) [33]; Figure S6: Graph regarding bias assessment of analytic prospective cohort studies using the Newcastle-Ottawa Scale (NOS); Figure S7: Graph regarding bias assessment of the retrospective case-control study using the Newcastle-Ottawa Scale (NOS); Figure S8: Graph regarding bias assessment of cross-sectional studies using the Joanna Briggs Institute scale (JBI); Figure S9: Graph regarding the assessment of bias in randomized clinical trials using the Risk of Bias scale (RoB 2); Figure S10: Forest-Plot and Odds Ratio results, 95% CI, allowing the comparison of the rate of peri-implantitis in patients with a history of periodontitis compared to healthy patients [13,14,24,26,27]; Figure S11: Forest-Plot and Odds Ratio results, 95% CI, allowing the comparison of the rate of peri-implantitis in patients with a history of severe chronic periodontitis compared to healthy patients [13,14,26,27]; Figure S12: Forest-Plot and Odds Ratio results, 95% CI, allowing the comparison of the rate of peri-implantitis in patients with a history of moderate chronic periodontitis compared to healthy patients [13,14,26,27]; Figure S13: Forest-Plot and Odds Ratio results, 95% CI, allowing the comparison of the rate of peri-implantitis in patients with a history of moderate and severe chronic periodontitis [13,26]; Figure S14: Forest-Plot and Odds Ratio results, 95% CI, allowing the comparison of the rate of peri-implantitis in patients with a history of chronic periodontitis compared to healthy patients [13,26]; Figure S15: Forest-Plot and Odds Ratio results, 95% CI, allowing the comparison of the rate of peri-implantitis in patients with a history of chronic and aggressive periodontitis [13,26]; Figure S16: Forest-Plot and Odds Ratio results, 95% CI, allowing the comparison of the survival rate in patients with a history of periodontitis compared to healthy patients [13,15,21,22,23,24,25,26]; Figure S17: Forest-Plot and Odds Ratio results, 95% CI, allowing the comparison of the survival rate in patients with a history of aggressive periodontitis compared to healthy patients [13,15,21,23,25,26]; Figure S18: Forest-Plot and Odds Ratio results, 95% CI, allowing the comparison of the survival rate in patients with a history of moderate chronic periodontitis compared to healthy patients [13,15,21,23,25,26]; Figure S19: Forest-Plot and Odds Ratio results, 95% CI, allowing the comparison of the survival rate in patients with a history of severe chronic periodontitis compared to healthy patients [22,24]; Figure S20: Forest-Plot and Odds Ratio results, 95% CI, allowing the comparison of the survival rate in patients with a history of moderate and severe chronic periodontitis [13,23,26]; Figure S21: Forest-Plot and Odds Ratio results, 95% CI, allowing the comparison of the survival rate in patients with a history of chronic periodontitis compared to healthy patients [13,23,26]; Figure S22: Forest-Plot and Odds Ratio results, 95% CI, allowing the comparison of the survival rate in patients with a history of chronic and aggressive periodontitis [13,23,26]; Figure S23: Forest-Plot and results of the standardized difference in means and its standard error, concerning alveolysis, between patients with treated chronic periodontitis and healthy patients [13,21,27]; Figure S24: Forest-Plot and results of the standardized difference in means and its standard error, concerning alveolysis, between patients with treated moderate chronic periodontitis and treated severe chronic periodontitis [13]; Figure S25: Forest-Plot and results of Hedges’s “g” and its standard deviation, for the different groups of periodontitis compared to the corresponding healthy group [13,15,22,26,27]; Figure S26: Forest-Plot and results of the standardized difference in means and its standard error, concerning PPD, in patients with a history of aggressive periodontitis compared to healthy patients [13,15,26,27]; Figure S27: Forest-Plot and results of the standardized difference in means and its standard error, concerning PPD, in patients with a history of moderate chronic periodontitis compared to healthy patients [13,15,26,27]; Figure S28: Forest-Plot and results of the standardized difference in means and its standard error, concerning PPD, in patients with a history of severe chronic periodontitis compared to healthy patients [22]; Figure S29: Forest-Plot and results of the standardized difference in means and its standard error, concerning PPD, in patients with a history of severe and moderate chronic periodontitis [13,26]; Figure S30: Forest-Plot and results of the standardized difference in means and its standard error, concerning PPD, in patients with a history of chronic periodontitis, whatever the severity, compared to healthy patients [13,26]; Figure S31: Forest-Plot and results of the standardized difference in means and its standard error, concerning PPD, in patients with a history of aggressive and chronic periodontitis [13,26]; Table S1: Studies excluded of PRISMA selection during the stage of eligibility with their exclusion criteria [35,36,37,38,39,40,41,42,43,44,45,46]; Table S2: Characteristics of included studies [13,14,15,16,21,22,23,24,25,26,27,28,29,30,31,32,33,34].
