# Supplementary material for: Implant Health in Treated Periodontitis Patients: A Systematic Review and Meta-Analysis
Source: Dent J (Basel). 2024 Jul 29;12(8):240. doi: 10.3390/dj12080240 (PMC11352642; doi:10.3390/dj12080240)
Supplement: Supplementary file 1 [file dentistry-12-00240-s001.zip › dentistry-3010671-supplementary.pdf]

SUPPLEMENTARY MATERIAL

Supplementary Table S1. Studies excluded of PRISMA selection during the stage of eligibility with their exclusion criteria [35–46].

NAME OF THE STUDY	TYPE OF STUDY	EXCLUSION CRITERIA
AGLIETTA et al. 2011 [35]	Analytical retrospective cohort study	Similar database to a study already included
MATARASSO et al. 2011 [36]	Analytical retrospective cohort study	Similar database to a study already included
HORWITZ et al. 2012 [37]	Descriptive prospective cohort study	Immediate implantation
ORMIANER et al. 2012 [38]	Analytical retrospective cohort study	Immediate implantation
AGUIRRE et al. 2013 [39]	Analytical prospective cohort study	Only 1 year of loading, follow-up time too short
APATZIDOU et al. 2017 [40]	Analytical retrospective cohort study	N<15 study power too low
CORREIA et al. 2017 [41]	Analytical retrospective cohort study	Attrition of 25% ≥ 20% (adequate attrition rate)
ZEZA et al. 2017 [42]	Analytical prospective cohort study	Attrition of 40% ≥ 20% (adequate attrition rate)
ALTAY et al. 2018 [43]	Pilot study	N<15 study power too low
SUNG et al. 2018 [44]	Descriptive transversal study	Patients diagnosed with active periodontitis during the study
GALLEGO et al. 2018 [45]	Descriptive retrospective cohort study	Attrition of 75% ≥ 20% (adequate attrition rate)
LIU et al. 2019 [46]	Case-control study	Immediate implantation

Supplementary Table S2. Characteristics of included studies [13–16,21–34].

**Study (follow-up)**	**Population characteristics**	**Intervention characteristics**	**Outcome measures**	**Funding**
	-Sample size (diagnosis)<br>-Age<br>-Recruitment<br>-Number of center	-Number of implants<br>-Implant type<br>-Surface treatment<br>-Regular maintenance	-Peri-implantitis rate (%)<br>-Survival rate (%)<br>-Mean of pocket depth (mm)<br>-Mean of alveolysis (mm)	
**QUANTITATIVE SYNTHESIS**				
CASADO et al. 2013<br>(8 years) [14]	-N=215 (CP)<br>-55 ±12.5<br>-University based<br>-1 center	-N=754<br>-NR<br>-NR<br>-Yes	-CP 59% / H 27%<br>-NR<br>-CP 2.1 ± 1.21 /<br>H 1.74 ± 0.88<br>- CP 2.23 mm ± 2.55<br>H 1.70 mm ± 2.16	NR
CHO-YAN LEE et al. 2012<br>(14 years) [27]	-N=30 (CP) / N=30 (H)<br>-57.36±7.28 (CP)-<br>58.28±6.60 (H)<br>-Private practice<br>-1 center	-N=117<br>-Straumann<br>-SLA/ TPS<br>-Yes	-CP 26.7% / H 13.1%<br>-NR<br>- CP 2.83 ± 0.59<br>H 2.81 ± 0.49<br>- CP 0.45 ± 0.94<br>H 0.26 ± 0.72	Non-governmental funding
GRAETZ et al. 2017	-N=29 (CP) / N=29 (H)<br>-23 to 76	-N=145<br>-NR	-NR	Self-funding

(10 years) [15]	-Hospital and university based<br>-2 centers	-NR<br>-Yes	- 5 y. PC 97.1% /<br>H 97.4%<br>10 y. PC 92.5% /<br>H 91.4%<br>-CP4.2±1.6mm<br>H2.9±0.8mm<br>CP18.7±18.2%<br>H12.5±21.3%	and Kiel University
JIANG et al.2013<br>(2 years) [25]	-N=30 (CP) / N=30 (H)<br>-18 to 50<br>-NR<br>-NR	-N=276<br>-NR<br>-NR<br>-Yes	-NR<br>- CP 95.97 % H 97.60 %<br>-NR<br>-NR	Governme ntal funding
LEVIN et al.2011<br>(12 years) [23]	-N=149 (PCM) / N=285 (PCS) / 283 (H)<br>-51.13 ± 12.35<br>- Hospital and university based / Clinical practice<br>-1 center	-N=2259<br>-NR<br>-NR<br>-Yes	-NR<br>-MCP 96.6% SCP 94.8% /<br>H 96.9%<br>- NR<br>- NR	Self-funding
RASPERINI et al. 2014<br>(10 years) [21]	-N=60 (CP) / N=60 (H)<br>- Sm/H (TS 51.2 ± 2.39 / TPS 51.5 ± 2.68)<br>Sm/PC (TS 51.3 ± 3.23/ TPS 51.7 ± 3.5)<br>NSm/H (ST 47.5 ± 2.9 / TPS 48.1 ± 2.1) NSm/PC (ST 47.2 ± 1.4 / TPS 46.5 ± 1.7)<br>-Hospital and university based / Private practice<br>-2 centers	-N=120<br>-Branemark/ Straumann<br>-MS / TPS<br>-Yes	-NR<br>- CP S 85%<br>CP NS 90%<br>H 95% (S et NS)<br>- NR<br>-2.28 ± 0.72	Departmen t university funding
ROCCUZZO et al. 2010- 2012 (10 years) [13]	- N=80 (CP) / N=32 (H)<br>- SCP 44±8.6 / MCP 49±15.3 / H 45±13<br>-Private practice<br>-1 center	-N=246<br>-Straumann<br>-TPS<br>-Yes	-H 10.7%<br>MCP 27% SCP 47.2%<br>-H 96.6%<br>MCP 92.8%<br>SCP 90%<br>-H 3.1±0.5 MPC 3.5±0.9 SCP 3.9±0.7<br>-H 0.75±0.88 MPC 1.14±1.11 SCP 0.98±1.22	NR
ROCCUZZO et al. 2014 (10 years) [26]	-N=91 (CP) / N=32 (H)<br>-MCP 53.3 ± 10.7/<br>SCP 52.7 ± 8.4/<br>H 43.3±12.4<br>-Private clinic of periodontology -1 center	-N=252<br>-Straumann<br>-SLA<br>-Yes	- H 18.8% MPC 2.2% SCP 66.7%<br>-H 100% MCP 96.9% SCP 97.1%<br>-MCP 4.6±1.3 SCP 4.8±1.4<br>H 4.4±1.1 -NR	NR
SWIERKOT et al. 2012<br>(16 years) [24]	-N=35 (AP) / N=18 (H)<br>-20 to 56 years<br>-Hospital and university based<br>-1 center	-N=179<br>-Branemark (MKII) Nobel Biocare, Osseotite BIOMET 3i -MS -Yes	-H 10% AP 26%<br>-H 100%<br>AP 96%<br>-NR-NR	No extern funding
THÖNE-MÜHLING et al. 2016<br>(4 years) [22]	-N=35 (AP) / N=18 (H)<br>-27 to 56 years (AP) /<br>25 to 57 years (H)<br>-Faculty of dental medicine	-N=179<br>- Branemark (MK II) Nobel Biocare, Osseotite Biomet 3i<br>-MS -Yes	-NR<br>-AP 97.3%<br>H 100%<br>-AP 3.50 ± 0.70 H 3.42 ± 0.81 -NR	No conflict of interest with financial

	-1 center			organizatio<br>n

Table 2. (Continued)

**Study (follow-up)**	**Population characteristics**	**Intervention characteristics**	**Outcome measures**	**Funding**
	-Sample size (diagnosis)<br>-Age<br>-Recruitment<br>-Number of centers	-Number of implants<br>-Implant type<br>-Surface treatment<br>-Regular maintenance	-Peri-implantitis rate (%)<br>-Survival rate (%)<br>-Mean of pocket depth (mm)<br>-Mean of alveolysis (mm)	
**QUALITATIVE SYNTHESIS**				
AGUIRRE et al. 2015<br>(17 years) [16]	-N=69 (AP)/N=170 (CP)<br>-53 years (49-79)<br>- Private clinic of periodontology<br>-1 center	-N=786<br>- TiOBlast Astra Tech/<br>TiUnite Nobel Biocare/<br>Steri-Oss<br>-Sandblasted/<br>Anodic oxidation<br>-Yes	-NR<br>-NR<br>-NR<br>-4.3mm±1.9	NR
DI GUARNIERI et al. 2020<br>(10 years) [30]	-N=58 (CP)<br>-55 years<br>-Private clinic of periodontology<br>-1 center	-N=127<br>-NR<br>-NR<br>-Yes	-NR<br>-90%<br>-NR<br>-3.1±1.2 mm	NR
MEYLE et al. 2014<br>(10 years) [29]	-N=20 (CP)<br>-48.7 ± 8.9 years<br>-Hospital and university based<br>-1 center	-N=54<br>-Frialit 2-Dentsply<br>-SLA<br>-Yes	- 23.8%<br>-96.3%<br>-3.3±1.0<br>-0.63±0.26mm	Self-funding and no extern founding
PANDOLFI et al. 2020<br>(10 years) [31]	-N=475 (CP)<br>-≥61 years 15.8%<br>≤60 years 84.2%<br>-Private clinic<br>-1 center	-N=1991<br>-Straumann<br>-SLA<br>-Yes	-12.9%<br>-96.1%<br>-NR<br>-NR	Self-funding
RAES et al. 2018<br>(5 years) [33]	-N=18-3=15 (AP)<br>-64 years (46 à 72)<br>- Hospital and university based<br>-1 center	-N=84<br>-Branemark MK III<br>-MS / TiUnite (oxyded surface)<br>-Yes	- Tur 7.14% TiU 28.57%<br>-Tur 97.6% TiU 100%<br>-Tur 3.1±1.0mm<br>TiU 4.2±2.6mm<br>- Tur 1.0±0.9mm<br>TiU 1.7±1.7mm	Funding by dentsply Sirona, Intra-Lock et Nobel Biocare and by the hospital-university
SAYARDOUST et al. 2013<br>(5 years) [28]	-N=80 (CP)<br>-53.5-54.2 years (Sm)<br>59.8-63.2 years (NSm)<br>-Hospital and university based<br>-1 center	-N=252<br>-Branemark<br>-MS/ TiUnite<br>-Yes	-NR<br>-92.9%<br>-NR<br>-S 1.39mm (1.57)<br>NS : 1.01mm (1.09)	Departmental funding
VAGIA et al. 2021<br>(10 years) [32]	-N=86<br>-66 years<br>-Hospital and university based	-N=260<br>-Straumann<br>-NR<br>-Yes	-12,8%<br>-NR<br>-NR<br>-NR	Self-funding

	-1 center			
XU et al. 2023 (5 years) [34]	-N=1528<br>-42.03 ± 15.00<br>-Hospital and university based<br>-1 center	-N=2998<br>-Nobel Biocare NAI<br>-Tapered-thread<br>-Yes	-NR<br>-95,39%<br>-NR<br>-NR	National Natural Science Foundation of China – Key Research and Development

Legends: AP: Aggressive Periodontitis; CP: Chronic Periodontitis; H: Health; MS: machined surface; MCP: Moderate Chronic Periodontitis; NR: not reported; NSm: Non Smokers; Sm: Smokers; SCP: Severe Chronic Periodontitis; SLA: Sand blast, Large grit, Acid-etch (implant surface); TPS: titanium plasma sprayed (implant surface)

Supplementary Figure S1. Graph regarding bias assessment of analytic retrospective cohort studies [14,15,21,22] using the Newcastle-Ottawa Scale (NOS)

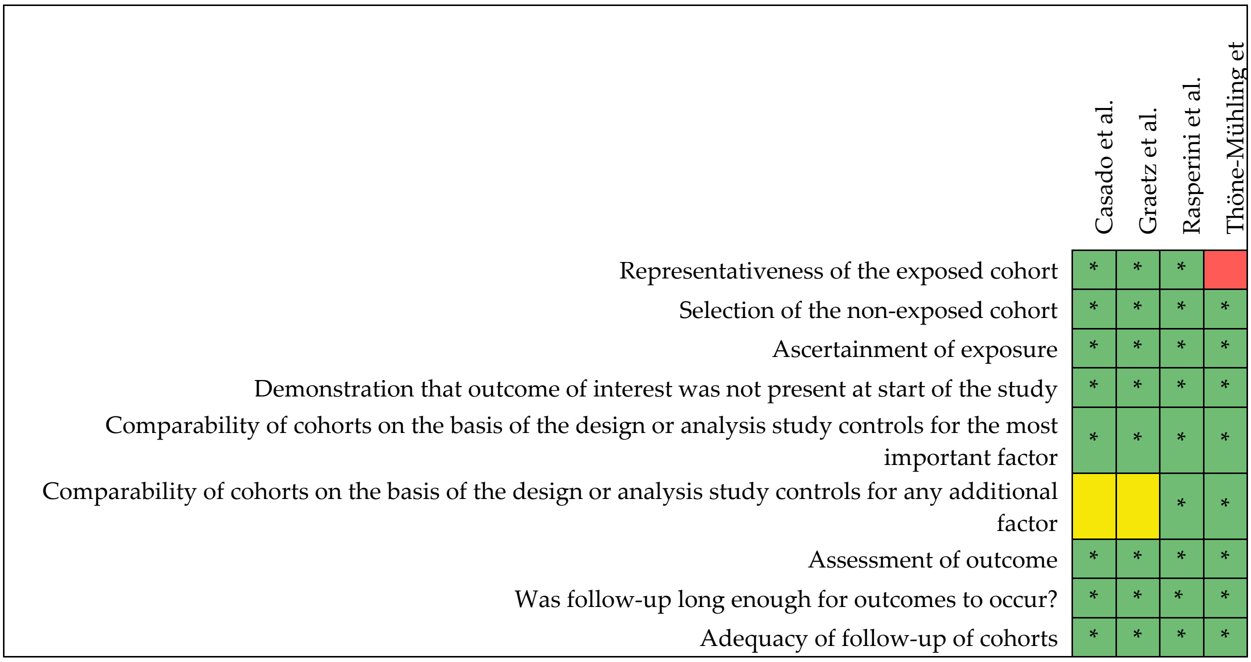

Supplementary Figure S2. Graph regarding bias assessment of analytic prospective cohort studies [13,23–26] using the Newcastle-Ottawa Scale (NOS)

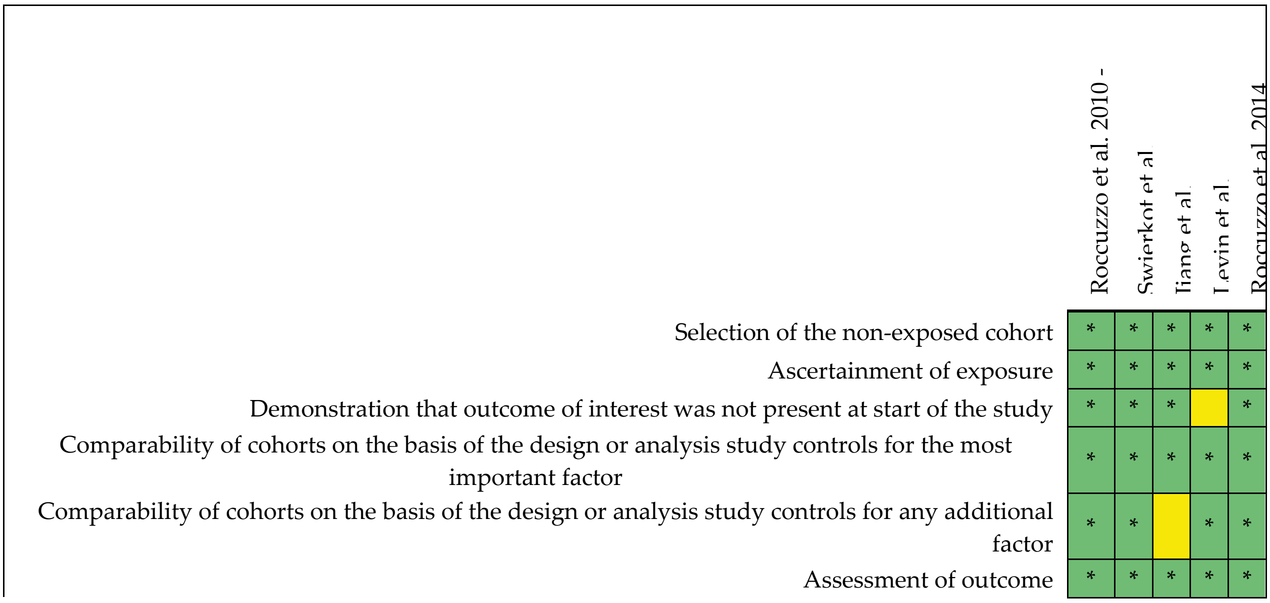

Was follow-up long enough for outcomes to occur?	*	*	*	*	*
Adequacy of follow-up of cohorts	*	*	*	*	*

Supplementary Figure S3. Graph regarding bias assessment of the retrospective case-control study [27,32,34] using the Newcastle-Ottawa Scale (NOS)

	Cho-Yan Lee et al.	Vagia et al.	Xu et al.
Is the case definition adequate	*	*	*
Representativeness of the cases	*	*	*
Selection of controls	*	*	*
Definition of controls	*	*	*
Comparability of cases and controls on the basis of the basis of design or analysis study controls for the most important factor	*	*	*
Comparability of cases and controls on the basis of the basis of design or analysis study controls for any additional factor	*	*	*
Ascertainment of exposure	*	*	*
Same method of ascertainment for cases and controls	*	*	*
Non –Response rate	*	*	*

	Aguirre et al. 2014	Guarnieri et al.	Pandolfi et al.	Sayardoust et al.	Meyle et al.
Were the criteria for inclusion in the sample clearly defined?	*	*	*	*	*
Were the study subjects and the setting described in detail?			*	*	*
Was the exposure measured in a valid and reliable way?	*	*	*	*	*
Were objective, standard criteria used for measurement of the condition?	*	*	*	*	*
Were confounding factors identified?				*	*
Were strategies to deal with confounding factors stated?	*	*	*	*	*
Were outcomes measured in a reliable way?	*	*	*	*	*
Was appropriate statistical analysis used?	*	*	*	*	*

Supplementary Figure S4. Graph regarding bias assessment of cross-sectional studies [16,28–31] using the Joanna Briggs Institute scale (JBI)

Supplementary Figure S5. Graph regarding the assessment of bias in randomized clinical trials [33] using the Risk of Bias scale (RoB 2)

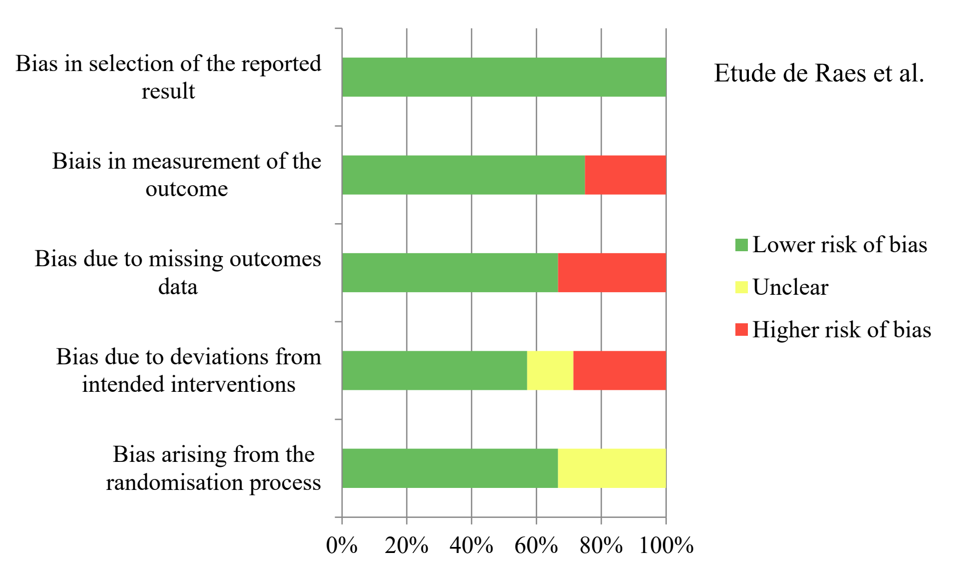

Supplementary Figure S6. Results of heterogeneity tests, regarding the peri-implantitis rate of studies included in the quantitative synthesis.

Model		Heterogeneity			
Model	Number Studies	Q-value	df (Q)	P-value	I-squared
Random	7	2,436	6	0,876	0,000

Supplementary Figure S7. Results of heterogeneity tests, regarding the survival rate of studies included in the quantitative synthesis.

Model		Heterogeneity			
Model	Number	Q-value	df (Q)	P-value	I-squared
Random	14	6,981	13	0,903	0,000

Supplementary Figure S8. Results of heterogeneity tests, regarding the alveolysis mean of studies included in the quantitative synthesis

Model		Heterogeneity			
Model	Number	Q-value	df (Q)	P-value	I-squared
Random	7	42,967	6	0,000 *	86,036

Supplementary Figure S9. Results of heterogeneity tests, regarding the alveolysis mean of studies included in the quantitative synthesis.

Model		Heterogeneity			
Model	Number	Q-value	df (Q)	P-value	I-squared
Random	7	28,671	6	0,000 *	79,073

Supplementary Figure S10. Forest-Plot and Odds Ratio results, 95% CI, allowing the comparison of the rate of peri-implantitis in patients with a history of periodontitis compared to healthy patients [13,14,24,26,27].

Study name	Subgroup within study	Odds ratio	Lower limit	Upper limit	p-Value	Relative weight
Roccuzzo 2010	PCM	3,086	0,761	12,519	0,115	7,30
Roccuzzo 2010	PCS	7,456	1,905	29,186	0,004 *	7,68
Cho-Yan Lee	PC	4,750	1,584	14,245	0,005 *	11,86
Swierkot	PAG	6,000	1,193	30,174	0,030 *	5,48
Casado	PC	3,951	2,215	7,047	0,000 *	42,72
Roccuzzo 2014	PCM	4,727	1,639	13,639	0,004 *	12,74
Roccuzzo 2014	PCS	8,667	2,935	25,588	0,000 *	12,21
		4,798	3,287	7,004	0,000 *	

Odds ratio and 95% CI

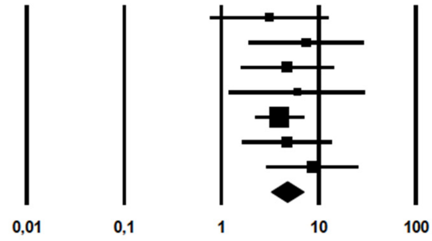

*p-Value < 0.05: significant difference

Legends: PAG: Generalized Aggressive Periodontitis; PC: Chronic Periodontitis; PCM: Moderate Chronic Periodontitis; PCS: Severe Chronic Periodontitis

Supplementary Figure S11. Forest-Plot and Odds Ratio results, 95% CI, allowing the comparison of the rate of peri-implantitis in patients with a history of chronic periodontitis compared to healthy patients [13,14,26,27].

Study name	Subgroup within study	Odds ratio	Lower limit	Upper limit	p-Value
Cho-Yan Lee	PC vs S	4,750	1,584	14,245	0,005 *
Casado	PC vs S	3,951	2,215	7,047	0,000 *
Roccuzzo 2010	PCM + PCS vs S	4,891	1,349	17,740	0,016 *
Roccuzzo 2014	PCM + PCS vs S	6,324	2,371	16,873	0,000 *
		4,550	2,965	6,981	0,000 *

Odds ratio and 95% CI

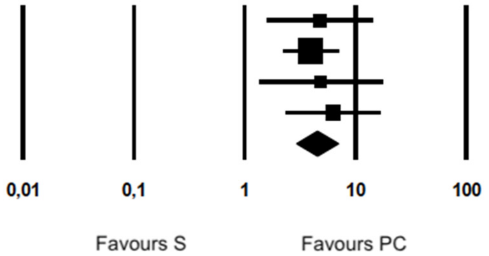

*p-Value < 0.05: significant difference

Legends: PC: Chronic Periodontitis; PCM: Moderate Chronic Periodontitis; PCS: Severe Chronic Periodontitis; S: Healthy Patient [13]

Supplementary Figure S12. Forest-Plot and Odds Ratio results, 95% CI, allowing the comparison of the rate of peri-implantitis in patients with a history of chronic and aggressive periodontitis [13,14,26,27].

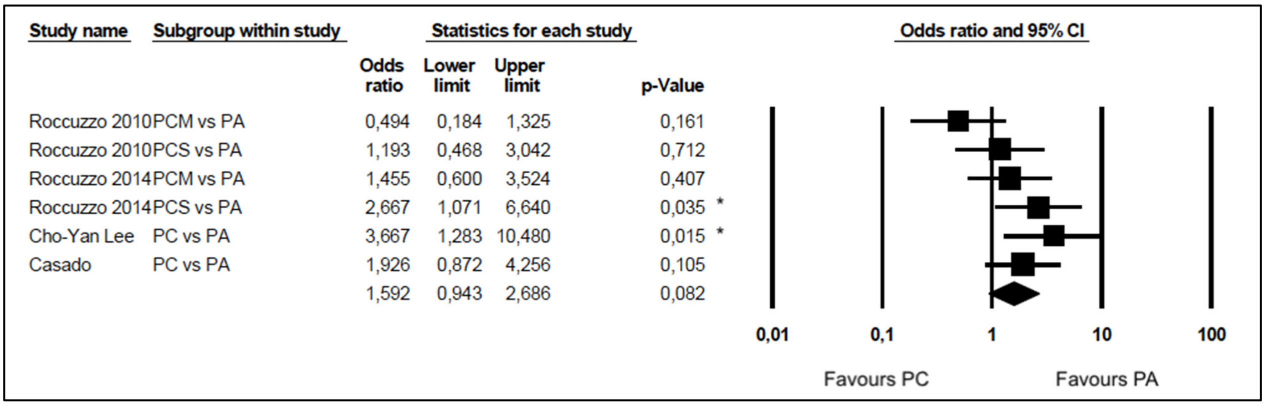

*p-Value < 0.05: significant difference

Legends: PA: Aggressive Periodontitis; PC: Chronic Periodontitis; PCM: Moderate Chronic Periodontitis; PCS: Severe Chronic Periodontitis

Supplementary Figure S13. Forest-Plot and Odds Ratio results, 95% CI, allowing the comparison of the rate of peri-implantitis in patients with a history of severe chronic periodontitis compared to healthy patients [13,26].

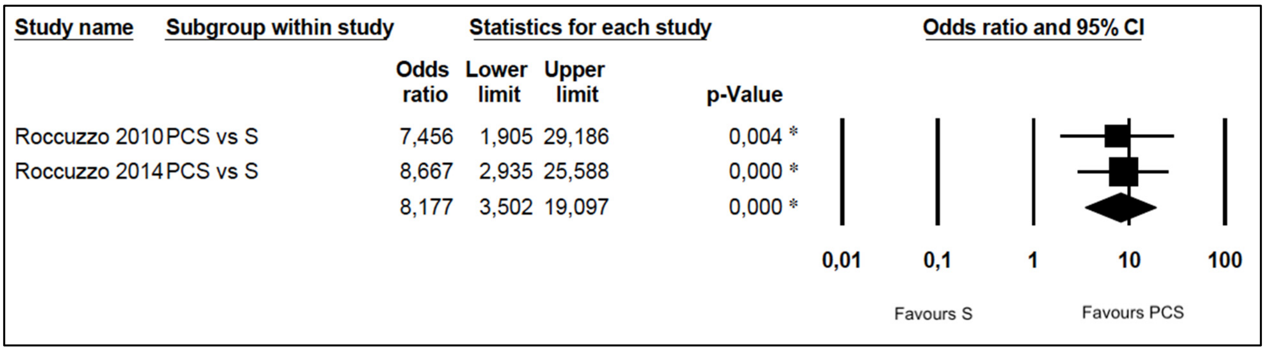

*p-Value < 0.05: significant difference

Legends: PCS: Severe Chronic Periodontitis; S: Healthy Patient

Supplementary Figure S14. Forest-Plot and Odds Ratio results, 95% CI, allowing the comparison of the rate of peri-implantitis in patients with a history of moderate chronic periodontitis compared to healthy patients [13,26].

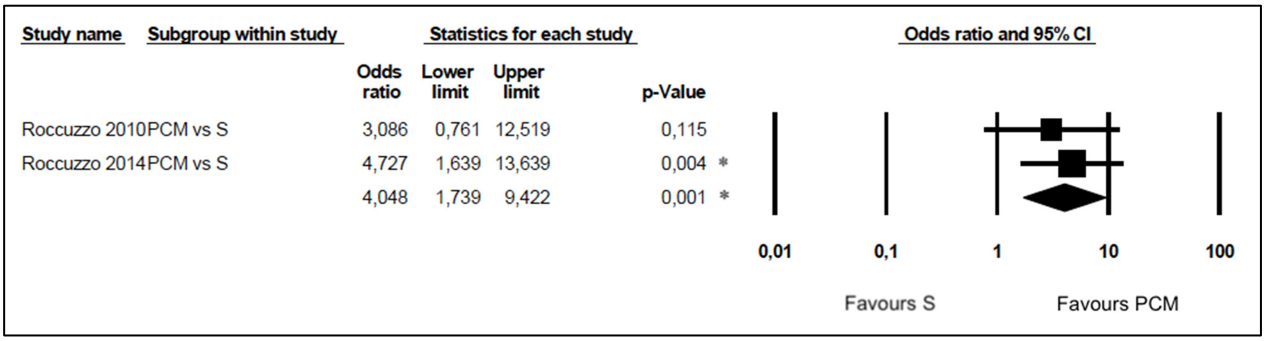

*p-Value < 0.05: significant difference

Legends: PCM: Moderate Chronic Periodontitis; S: Healthy Patient

Supplementary Figure S15. Forest-Plot and Odds Ratio results, 95% CI, allowing the comparison of the rate of peri-implantitis in patients with a history of moderate and severe chronic periodontitis [13,26].

Study name	Subgroup within study	Odds ratio	Lower limit	Upper limit	p-Value
Roccuzzo 2010	PCM vs PCS	0,414	0,156	1,100	0,077
Roccuzzo 2014	PCM vs PCS	0,545	0,234	1,273	0,161
		0,484	0,255	0,919	0,027 *

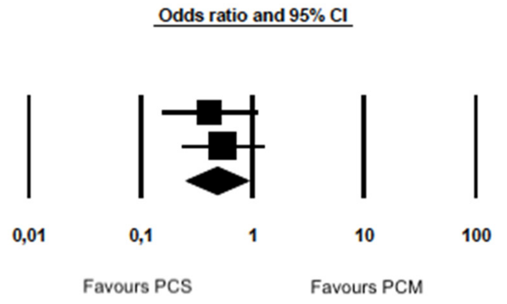

*p-Value < 0.05: significant difference

Legends: PCM: Moderate Chronic Periodontitis; PCS: Severe Chronic Periodontitis

Supplementary Figure S16. Forest-Plot and Odds Ratio results, 95% CI, allowing the comparison of the survival rate in patients with a history of periodontitis compared to healthy patients [13,15,21–26].

Study name	Subgroup within study	Odds ratio	Lower limit	Upper limit	p-Value
Roccuzzo 2010	PCM	0,426	0,086	2,123	0,298
Roccuzzo 2010	PCS	0,305	0,064	1,464	0,138
Levin	PCM	0,915	0,472	1,772	0,792
Levin	PCS	0,583	0,355	0,958	0,033 *
Swierkot	PAG	0,064	0,004	1,079	0,057
Jiang	PC	0,577	0,141	2,354	0,443
Rasperini	PC Sm/TPS	0,162	0,007	3,847	0,260
Rasperini	PC Sm/MS	1,000	0,054	18,574	1,000
Rasperini	PC NSm/TPS	0,298	0,028	3,146	0,314
Rasperini	PC NSm/MS	1,000	0,058	17,181	1,000
Roccuzzo 2014	PCM	0,245	0,012	4,835	0,355
Roccuzzo 2014	PCS	0,261	0,013	5,143	0,377
Thöne-Mühling	PAG	0,530	0,028	10,104	0,673
Graetz	PC	0,774	0,265	2,258	0,639
		0,601	0,435	0,832	0,002 *

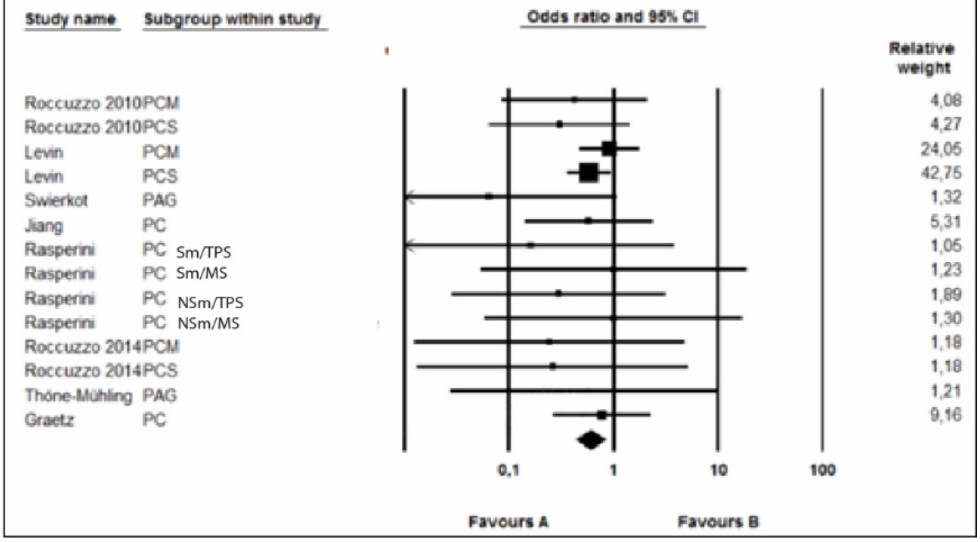

*p-Value < 0.05: significant difference

Legends: PAG: Generalized Aggressive Periodontitis; PC: Chronic Periodontitis; PCM: Moderate Chronic Periodontitis; PCS: Severe Chronic Periodontitis; NSm: Non-Smokers; Sm: Smokers; MS: machined surface; TPS: titanium plasma sprayed (implant surface)

Supplementary Figure S17. Forest-Plot and Odds Ratio results, 95% CI, allowing the comparison of the survival rate in patients with a history of chronic periodontitis compared to healthy patients [13,15,21,23,25,26].

Study name	Subgroup within study	Odds ratio	Lower limit	Upper limit	p-Value
Graetz	PC vs S	0,774	0,265	2,258	0,639
Jiang	PC vs S	0,577	0,141	2,354	0,443
Levin	PCM + PCS vs S	0,654	0,405	1,057	0,083
Rasperini	PC vs S	0,398	0,098	1,621	0,199
Roccuzzo 2010	PCM + PCS vs S	0,358	0,080	1,604	0,179
Roccuzzo 2014	PCM + PCS vs S	0,272	0,015	4,899	0,377
		0,604	0,412	0,887	0,010 *

Statistics for each study

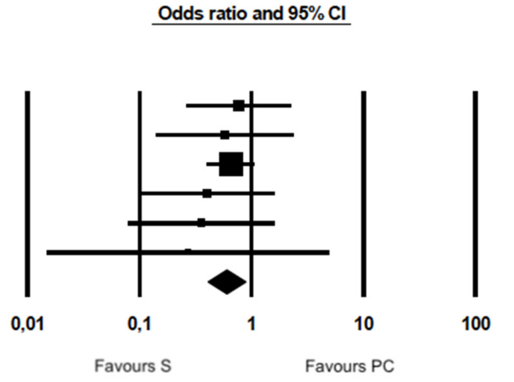

*p-Value < 0.05: significant difference

Legends: PC: Chronic Periodontitis; PCM: Moderate Chronic Periodontitis; PCS: Severe Chronic Periodontitis; S: Healthy Patient

Supplementary Figure S18. Forest-Plot and Odds Ratio results, 95% CI, allowing the comparison of the survival rate in patients with a history of chronic and aggressive periodontitis [13,15,21,23,25,26].

Study name	Subgroup within study	Odds ratio	Lower limit	Upper limit	p-Value
Roccuzzo 2010	PCM vs PA	0,437	0,161	1,181	0,102
Roccuzzo 2010	PCS vs PA	0,313	0,123	0,795	0,015 *
Levin	PCM vs PA	1,000	0,443	2,257	1,000
Levin	PCS vs PA	0,638	0,321	1,267	0,199
Jiang	PC vs PA	0,828	0,295	2,322	0,719
Rasperini	PC vs PA	0,263	0,096	0,721	0,009 *
Roccuzzo 2014	PCM vs PA	1,076	0,290	3,994	0,912
Roccuzzo 2014	PCS vs PA	1,146	0,309	4,248	0,839
Graetz	PC vs PA	0,265	0,100	0,698	0,007 *
		0,550	0,377	0,802	0,002 *

Statistics for each study

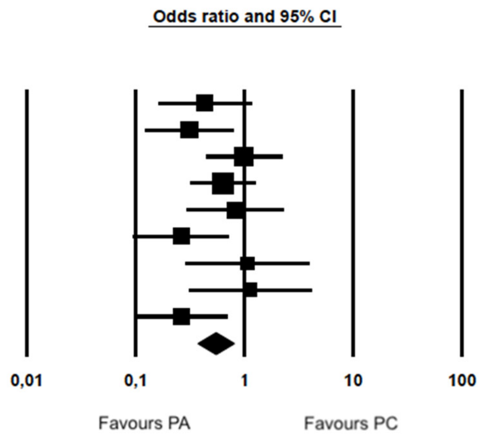

*p-Value < 0.05: significant difference

Legends: PA: Aggressive Periodontitis; PC: Chronic Periodontitis; PCM: Moderate Chronic Periodontitis; PCS: Severe Chronic Periodontitis

Supplementary Figure S19. Forest-Plot and Odds Ratio results, 95% CI, allowing the comparison of the survival rate in patients with a history of aggressive periodontitis compared to healthy patients [22,24].

Study name	Subgroup within study	Odds ratio	Lower limit	Upper limit	p-Value
Swierkot	PA vs S	0,362	0,020	6,596	0,493
Thöne-Mühling	PA vs S	0,530	0,028	10,104	0,673
		0,437	0,055	3,455	0,432

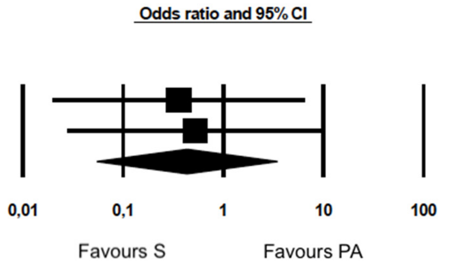

*p-Value < 0.05: significant difference

Legends: PA: Aggressive Periodontitis; S: Healthy Patient

Supplementary Figure S20. Forest-Plot and Odds Ratio results, 95% CI, allowing the comparison of the survival rate in patients with a history of moderate chronic periodontitis compared to healthy patients [13,23,26].

Study name	Subgroup within study	Odds ratio	Lower limit	Upper limit	p-Value
Roccuzzo 2010	PCM vs S	0,426	0,086	2,123	0,298
Roccuzzo 2014	PCM vs S	0,245	0,012	4,835	0,355
Levin	PCM vs S	0,915	0,472	1,772	0,792
		0,780	0,429	1,420	0,416

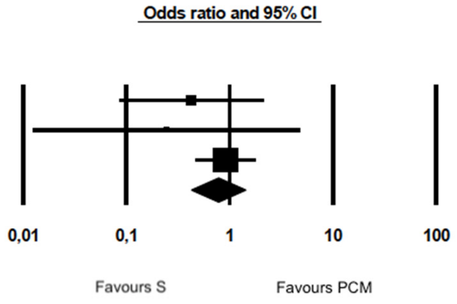

*p-Value < 0.05: significant difference

Legends: PCM: Moderate Chronic Periodontitis; S: Healthy Patient

Supplementary Figure S21. Forest-Plot and Odds Ratio results, 95% CI, allowing the comparison of the survival rate in patients with a history of severe chronic periodontitis compared to healthy patients [13,23,26].

Study name	Subgroup within study	Odds ratio	Lower limit	Upper limit	p-Value
Roccuzzo 2010	PCS vs S	0,305	0,064	1,464	0,138
Roccuzzo 2014	PCS vs S	0,261	0,013	5,143	0,377
Levin	PCS vs S	0,583	0,355	0,958	0,033 *
		0,540	0,339	0,861	0,010 *

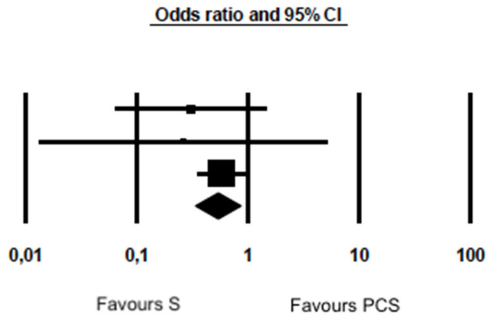

*p-Value < 0.05: significant difference

Legends: PCS: Severe Chronic Periodontitis; S: Healthy Patient

Supplementary Figure S22. Forest-Plot and Odds Ratio results, 95% CI, allowing the comparison of the survival rate in patients with a history of moderate and severe chronic periodontitis [13,23,26].

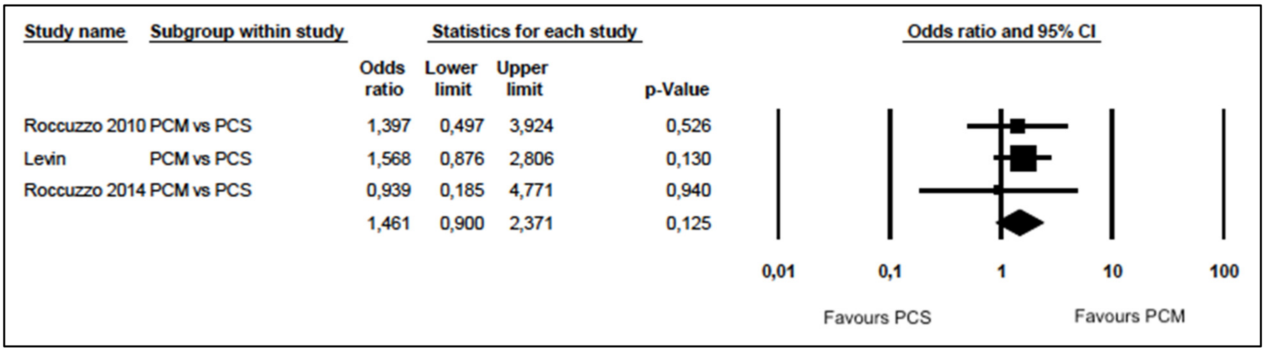

*p-Value < 0.05: significant difference

Legends: PCM: Moderate Chronic Periodontitis; PCS: Severe Chronic Periodontitis

Supplementary Figure S23. Forest-Plot and results of the standardized difference in means and its standard error, concerning alveolysis, between patients with treated chronic periodontitis and healthy patients [13,21,27].

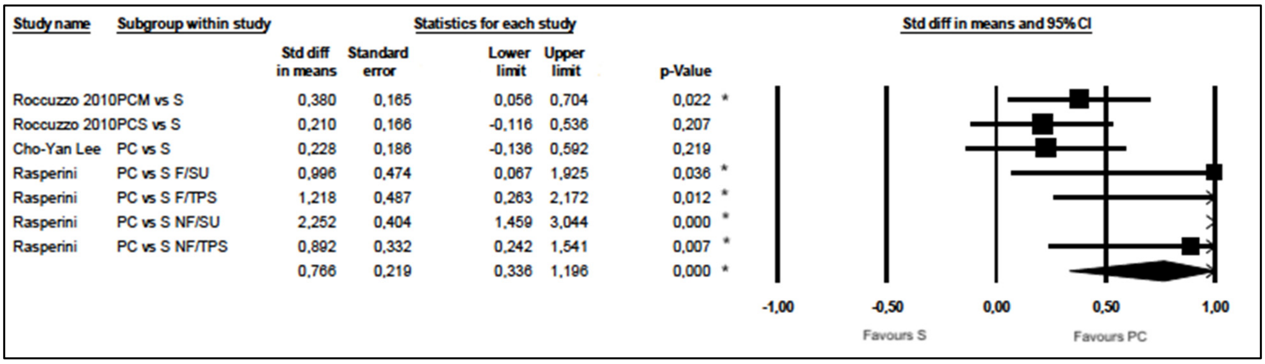

*p-Value < 0.05: significant difference

Legends: PC: Chronic Periodontitis; PCM: Moderate Chronic Periodontitis; PCS: Severe Chronic Periodontitis; S: Healthy Patient

Supplementary Figure S24. Forest-Plot and results of the standardized difference in means and its standard error, concerning alveolysis, between patients with treated moderate chronic periodontitis and treated severe chronic periodontitis [13].

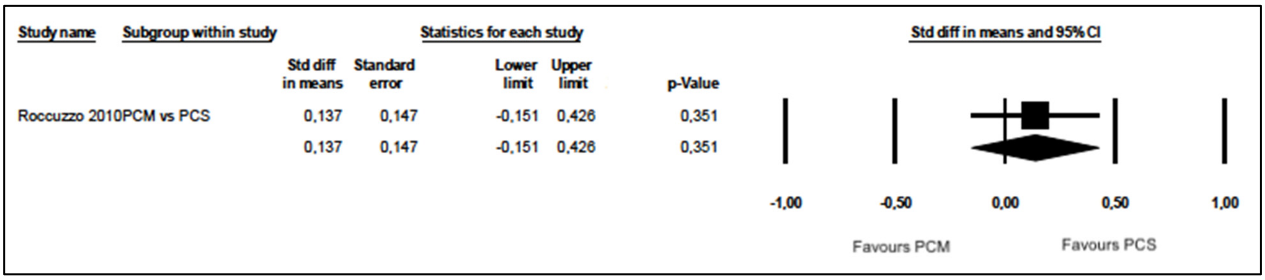

*p-Value < 0.05: significant difference

Legends: PCM: Moderate Chronic Periodontitis; PCS: Severe Chronic Periodontitis

Supplementary Figure S25. Forest-Plot and results of Hedges's "g" and its standard deviation, for the different groups of periodontitis compared to the corresponding healthy group [13,15,22,26,27].

Study name	Subgroup within study	Hedges's g	Standard error	Variance	Lower limit	Upper limit	p-Value
Roccuzzo 2010	PCM	0,517	0,166	0,028	0,192	0,843	0,002 *
Roccuzzo 2010	PCS	1,269	0,180	0,033	0,915	1,623	0,000 *
Cho-Yan Lee	PC	0,037	0,184	0,034	-0,324	0,397	0,841
Roccuzzo 2014	PCM	0,161	0,169	0,029	-0,171	0,494	0,341
Roccuzzo 2014	PCS	0,305	0,168	0,028	-0,025	0,635	0,070
Thöne-Mühling	PAG	0,111	0,199	0,040	-0,280	0,501	0,578
Graetz	PC	1,038	0,176	0,031	0,692	1,383	0,000 *
		0,493	0,179	0,032	0,142	0,844	0,006 *

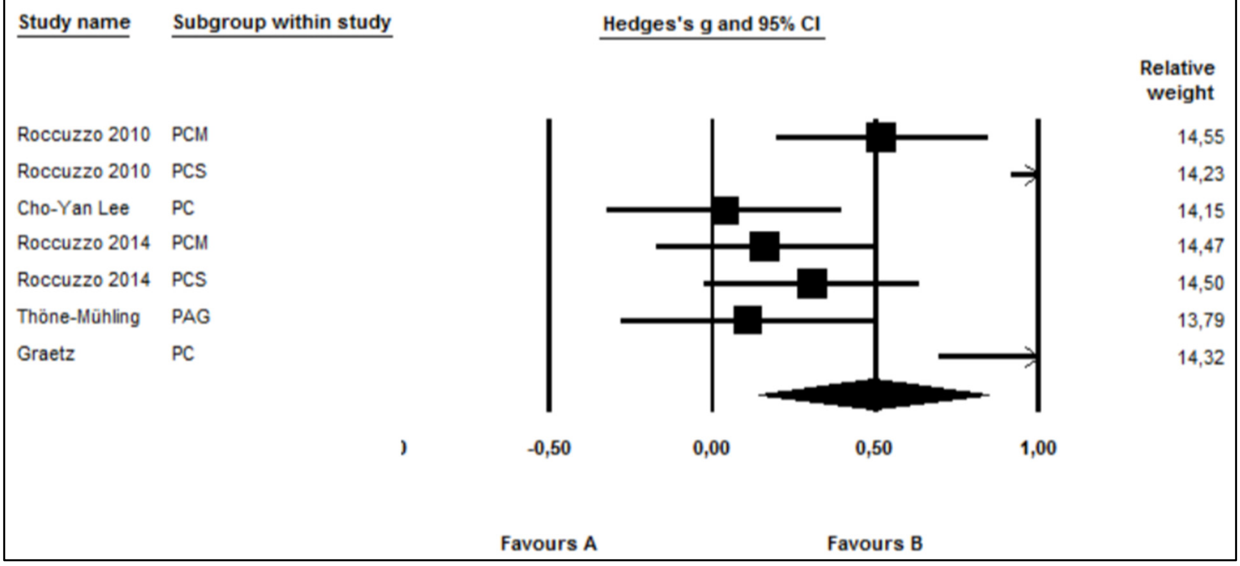

*p-Value < 0.05: significant difference

Legends: PAG: Generalized Aggressive Periodontitis; PC: Chronic Periodontitis; PCM: Moderate Chronic Periodontitis; PCS: Severe Chronic Periodontitis

Supplementary Figure S26. Forest-Plot and results of the standardized difference in means and its standard error, concerning PPD, in patients with a history of chronic periodontitis, whatever the severity, compared to healthy patients [13,15,26,27].

Study name	Subgroup within study	Std diff in means	Standard error	Lower limit	Upper limit	p-Value
Roccuzzo 2010	PCM vs S	0,520	0,167	0,193	0,847	0,002 *
Roccuzzo 2010	PCS vs S	1,276	0,181	0,920	1,631	0,000 *
Cho-Yan Lee	PC vs S	0,037	0,185	-0,326	0,400	0,841
Roccuzzo 2014	PCM vs S	0,162	0,170	-0,172	0,496	0,341
Roccuzzo 2014	PCS vs S	0,307	0,169	-0,025	0,638	0,070
Graetz	PC vs S	1,043	0,177	0,696	1,390	0,000 *
		0,556	0,199	0,167	0,946	0,005 *

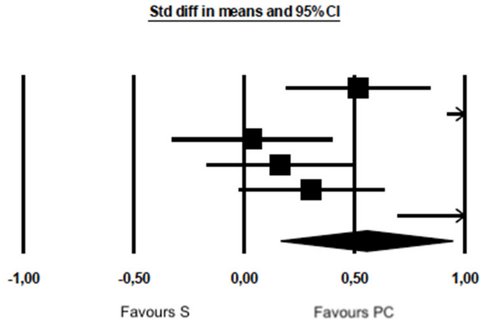

*p-Value < 0.05: significant difference

Legends: PC: Chronic Periodontitis; PCM: Moderate Chronic Periodontitis; PCS: Severe Chronic Periodontitis; S: Healthy Patient

Supplementary Figure S27. Forest-Plot and results of the standardized difference in means and its standard error, concerning PPD, in patients with a history of aggressive and chronic periodontitis [13,15,26,27].

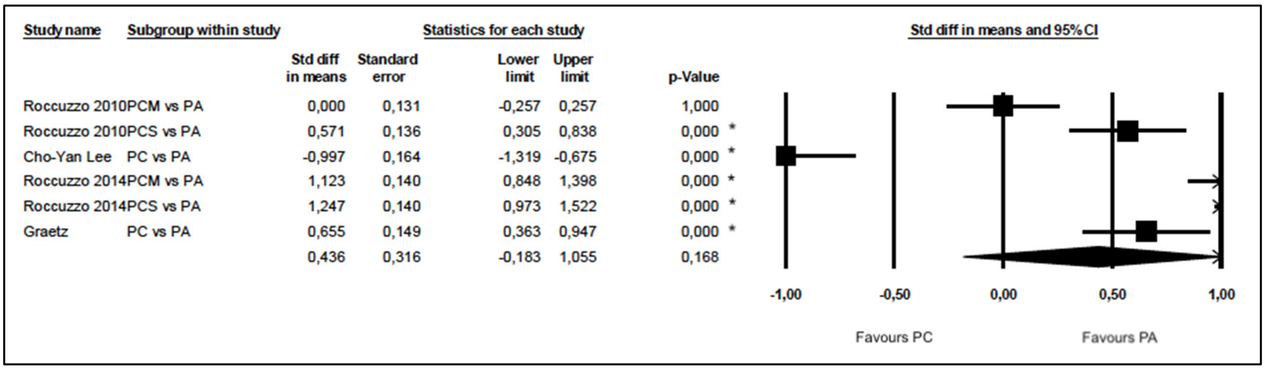

Study name	Subgroup within study	Std diff in means	Standard error	Lower limit	Upper limit	p-Value
Roccuzzo 2010	PCM vs PA	0,000	0,131	-0,257	0,257	1,000
Roccuzzo 2010	PCS vs PA	0,571	0,136	0,305	0,838	0,000 *
Cho-Yan Lee	PC vs PA	-0,997	0,164	-1,319	-0,675	0,000 *
Roccuzzo 2014	PCM vs PA	1,123	0,140	0,848	1,398	0,000 *
Roccuzzo 2014	PCS vs PA	1,247	0,140	0,973	1,522	0,000 *
Graetz	PC vs PA	0,655	0,149	0,363	0,947	0,000 *
		0,436	0,316	-0,183	1,055	0,168

*p-Value < 0.05: significant difference

Legends: PA: Aggressive Periodontitis; PC: Chronic Periodontitis; PCM: Moderate Chronic Periodontitis; PCS: Severe Chronic Periodontitis

Supplementary Figure S28. Forest-Plot and results of the standardized difference in means and its standard error, concerning PPD, in patients with a history of aggressive periodontitis compared to healthy patients [22].

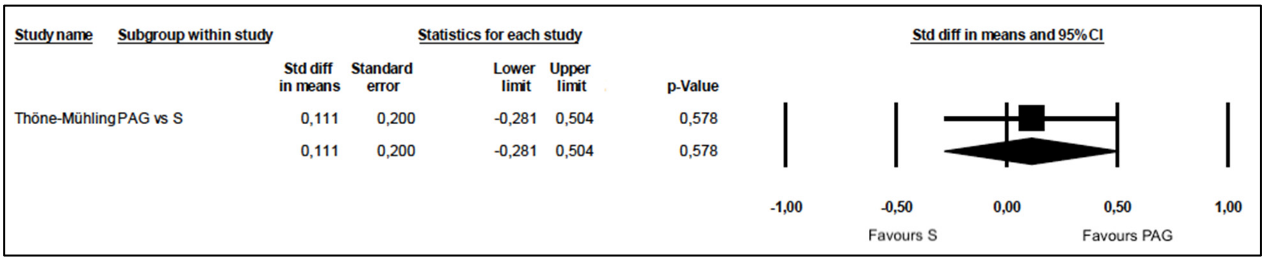

Study name	Subgroup within study	Std diff in means	Standard error	Lower limit	Upper limit	p-Value
Thöne-Mühling	PAG vs S	0,111	0,200	-0,281	0,504	0,578
		0,111	0,200	-0,281	0,504	0,578

*p-Value < 0.05: significant difference

Legends: PAG: Generalize Aggressive Periodontitis; S: Healthy Patient

Supplementary Figure S29. Forest-Plot and results of the standardized difference in means and its standard error, concerning PPD, in patients with a history of moderate chronic periodontitis compared to healthy patients [13,26].

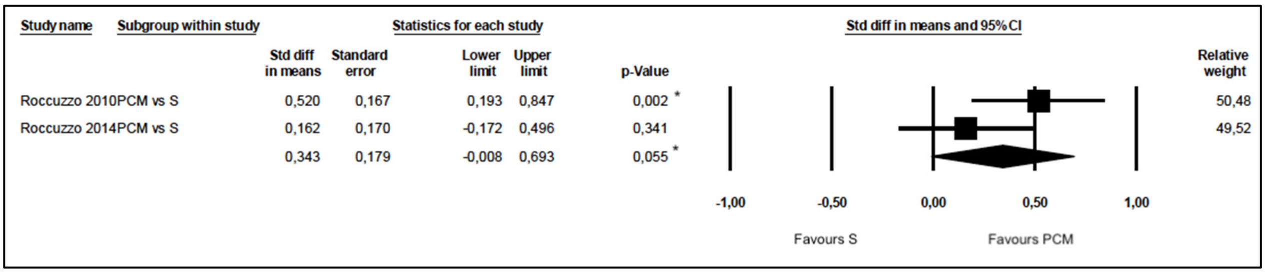

Study name	Subgroup within study	Std diff in means	Standard error	Lower limit	Upper limit	p-Value	Relative weight
Roccuzzo 2010	PCM vs S	0,520	0,167	0,193	0,847	0,002 *	50,48
Roccuzzo 2014	PCM vs S	0,162	0,170	-0,172	0,496	0,341	49,52
		0,343	0,179	-0,008	0,693	0,055 *	

*p-Value < 0.05: significant difference

Legends: PCM: Moderate Chronic Periodontitis; S: Healthy Patient

Supplementary Figure S30. Forest-Plot and results of the standardized difference in means and its standard error, concerning PPD, in patients with a history of severe chronic periodontitis compared to healthy patients [13,26].

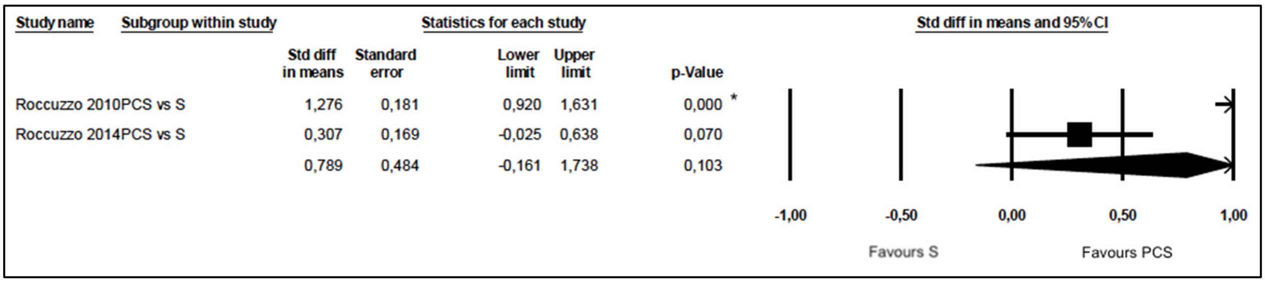

Study name	Subgroup within study	Std diff in means	Standard error	Lower limit	Upper limit	p-Value
Roccuzzo 2010	PCS vs S	1,276	0,181	0,920	1,631	0,000 *
Roccuzzo 2014	PCS vs S	0,307	0,169	-0,025	0,638	0,070
		0,789	0,484	-0,161	1,738	0,103

*p-Value < 0.05: significant difference
